# Prevention of Ventriculostomy Related Infection: Effectiveness of Impregnated Biomaterial

**DOI:** 10.3390/ijms24054819

**Published:** 2023-03-02

**Authors:** Sylvain Diop, Ariane Roujansky, Hatem Kallel, Roman Mounier

**Affiliations:** 1 Anesthesiology and Intensive Care Department, Marie Lannelongue Surgical Hospital, 133 Avenue de la Résistance, 92350 Le Plessis Robinson, France; 2 Intensive Care Unit, Cayenne General Hospital, Av des Flamboyants, 97306 Cayenne, France; 3 Tropical Biome and Immuno-Pathology CNRS UMR-9017, Inserm U 1019, University of French Guiana, 97300 Cayenne, France; 4 Department of Neuro-ICU, GHU-Paris, Paris University, 75014 Paris, France; 5 INSERM U955, Team 15, Biomedical Research Institute, University Paris-Est-Creteil (UPEC), 94000 Paris, France

**Keywords:** external ventricular drain, biofilm, bacteria, silver nanoparticles, antibiotics impregnated, biomaterial

## Abstract

External ventricular drain(EVD) exposes the patient to infectious complications which are associated with significant morbidity and economic burden. Biomaterials impregnated with various antimicrobial agents have been developed to decrease the rate of bacterial colonization and subsequent infection. While promising, antibiotics and silver-impregnated EVD showed conflicting clinical results. The aim of the present review is to discuss the challenges associated with the development of antimicrobial EVD catheters and their effectiveness from the bench to the bedside.

## 1. Introduction

Bacteria are among the oldest life form on earth [1]. The most ancient proof of their existence goes back to 3.8 billion years ago [1]. Over time they colonized the terrestrial environment and were exposed to extreme conditions [2,3]. Early in their evolution, bacteria acquired the capacity to produce biofilm (the most ancient trace of biofilm goes back around 3.2 and 3.4 billion years ago), a particular lifestyle able to link bacteria together and provide advantageous mechanisms of protection ensuring their survival against hostile environmental conditions [4]. Bacteria play an essential role in human health and development [4,5]. All parts of the human epithelium (skin, gut, lungs, among others) are colonized by a large diversity of bacteria, fungi, or viruses forming microbiota. Most of these bacteria live under biofilm conditions [4].

In the twentieth century major medical advances have been made regarding the development of long-term (prosthesis, pacemaker) or short-term (central venous catheter, EVD) implanted medical devices, revolutionizing the prognosis of many diseases [6]. Concomitantly, it raises the problematic of associated infectious complications. From several decays, bacteria adhesion, and biofilm formation at the surface of biomaterials have been incriminated as the main pathway of infection [7,8]. Consequently, manufacturers proposed new innovating materials which were expected to decrease the rate of bacterial colonization and infection. In this review, we focus on the effectiveness of EVD impregnated with antimicrobials agent (antibiotics or silver) to prevent ventriculostomy-related infection (VRI).

## 2. Background

EVD are widely used in neurosurgery to control cerebral hypertension mainly related to subarachnoid hemorrhage (SAH) or traumatic brain injury [9]. It consists of a catheter inserted through the skull into the ventricles by a neurosurgeon, allowing the drainage of the cerebrospinal fluid (CSF) and the monitoring and control of the intracranial pressure [9]. It also exposes the patient to VRI leading to higher morbidity and economic burden [10]. The pooled incidence of VRI is 11.4 per 1000 catheters per day and the main risk factors identified are SAH, intraventricular hemorrhage, and CSF leakage at the point of insertion [10]. Duration of EVD catheterization remained a debated risk factor, but in the majority of the studies, VRI occurred around day 10 [11,12]. The most frequent bacteria implicated in VRI are Gram-positive cocci (GPC) belonging to the head skin flora (*Staphylococcus epidermidis*, *Staphylococcus aureus*, *Staphylococcus* spp., *Streptococcus* spp.) [11]. It has been postulated that VRI involves prior colonization of the surface device by bacterial biofilm originating from the skin microbiota [13]. Periprocedural prophylactic and prolonged intravenous antibiotics failed to demonstrate convincing evidence to prevent VRI [11,14]. Consequently, EVD impregnated with an antimicrobial agent has been developed to reduce the risk of bacterial adhesion, biofilm formation, and VRI development.

## 3. Silver-Impregnated EVD

Currently, numerous medical devices impregnated or coated with silver nanoparticles have been developed such as urinary catheters, central venous catheters (CVC), or EVD. The most studied and worldwide available silver-impregnated EVD is the Silverline^TM^ EVD. The catheter is made in polyurethane recovered with 1% of silver nanoparticles and 1% of insoluble silver salt. According to the manufacturers (Spielberg KG), it allows a continuous release of silver ions with a broad-spectrum activity of up to 32 h [15].

### 3.1. Antimicrobial Effect of Silver

The antimicrobial properties of silver have been known for thousands of years. Ancient Egyptians were familiar with the use of various metals, such as lead or silver, to treat or prevent infectious diseases [16]. Silver has good biocompatibility with mammalian cells and a broad-spectrum antimicrobial activity against both GPC, Gram-negative rods (GNR), and fungi even at low concentrations. The antimicrobial action is mediated by the direct toxicity of silver ions on bacteria through several mechanisms such as the generation of reactive oxygen species, damage of intracellular structure and proteins, alteration of signal transduction pathway, or electron chain transport [16]. Silver ion has a high affinity to peptidoglycan. It seems less effective in GPC because the large peptidoglycan wall could prevent the silver ion from reaching the bacteria cytoplasm [17]. Silver ions also demonstrated an anti-biofilm activity in vitro against both GPC and GNR. The antibacterial activity is very short because, in vivo, the silver ion quickly binds an anion (such as chloride) and precipitates [18]. Thus, to be effective, silver must be released continuously from the biomaterial surface. The antimicrobial effect of silver nanoparticles also depends on several factors such as size, shape, colloidal state, and the concentration of silver ions generated over time [19]. It leads to the development of numerous impregnation methods on biopolymer allowing the release of a small concentration of silver ions over time.

### 3.2. Synthesis of Silver-Impregnated Biomaterial and Experimental Results

Numerous synthesis methods for silver nanoparticles are available such as chemical (i.e., chemical reduction, electrochemical synthesis, pyrolysis method) or physical methods (i.e., arc discharge, laser irradiation). Recently a green synthetic process has also been developed, limiting the use of toxic chemical compounds [18]. The challenge associated with the synthesis of silver nanoparticles-impregnated biomaterial is multi-faceted: It must allow the synthesis of small-size nanoparticles (<100 nm) homogeneously distributed along the catheter and released continuously at a predictable rate over time, without local or systemic toxic effect. Polyurethane is a biopolymer with wide application in the medical field because of its biocompatibility and its advantageous physical properties [20].

Saveleyev et al. experimentally tested the synthesis and effectiveness of silver nanoparticles-impregnated polyurethane catheters [20]. The nanoparticles synthesized had a spherical shape and a size of 10 to 110 nm without changing the nature of the biomaterial. The impregnated material demonstrated bactericidal and bacteriostatic activity against *S. aureus*, GNR (*Pseudomonas aeruginosa*, *Enterobacter aerogenes*, *Klebsiella pneumoniae*, *Proteus mirabilis*, and *Escherichia coli*), and fungi [20]. Another study tested the impregnation of silver nanoparticles on polyurethane CVC grafted with acrylic acids. Scanning electron microscopy (SEM) showed that silver nanoparticles had a mean size of 45 nm, however, the concentration of silver in the biomaterial was very low. The antimicrobial effect was observed against *E coli* and methicillin-resistant *S. aureus* (MRSA) strains [21]. The data from the Manufacturer showed that Silverline^TM^ catheters have antimicrobial activity on GPC, GNR, and *Candida* (with a concentration of 10^7^ to 10^8^ cfu/mL according to the strain considered) when assessed with the roll culture plate method [15].

Bayston et al. investigated specifically in vitro Silverline^TM^ EVD effectiveness against different strains of *Staphylococci* and *Escherichia coli* at a concentration of 10^4^ cfu/mL during in and out flow conditions and found a rapid decrease of the antimicrobial activity over time, presumably due to the large size and the low density of the silver nanoparticles. Indeed, SEM observation showed that the silver nanoparticles had a diameter of 500 nm and were not uniformly disposed onto the catheter. Silverline^TM^ EVD were unable to kill 100% of the bacteria attached during flow conditions [22]. The application of a conditioning film on the catheter, mimicking in vivo conditions did not influence the antimicrobial effect of the EVD [22].

Galiano et al. investigated the concentration of silver by atomic absorption spectroscopy on an artificial CSF fluid crossing continuously (10 mL per hour at 37 °C) polyurethane ventricular shunt (VS) impregnated with silver nanoparticles (Silverline^TM^) and sampled every 24 h. They found no silver in each sample and expressed concerns about the effective delivery of silver by the catheter over time [23]. Moreover, they found no difference in bacterial growth when *S. aureus* and *E. coli* strains were exposed to Silverline^TM^ and a control catheter [23].

## 4. Effectiveness of Silver-Impregnated EVD in Clinical Practice

In the Infectious Disease Society of America (IDSA) guidelines regarding the prevention of healthcare-associated ventriculitis and meningitidis, published in 2017, the use of antimicrobial-impregnated EVD is recommended, but Silverline^TM^ EVD are not specifically mentioned, whereas antibiotics-impregnated EVD (AI-EVD) are [10]. Results of the SILVER randomized clinical trial (RCT), including 325 patients, found a significant decrease in EVD infection with Silverline^TM^ compared to unprocessed EVD (12.3 % and 21.4%, *p* = 0.043; respectively). VRI was defined as bacteria identified on Gram stain or isolated by culture in a CSF sample [24]. Another RCT assessing the effectiveness of silver-impregnated lumbar drains compared to unprocessed drains included 48 patients and found a similar rate of infection-related devices in both groups (4.2% and 16.7%, *p* = 0.16; respectively). Infection was defined as a positive CSF culture or at least one sign of meningitidis and (1) increased CSF white blood cell count, proteins level, or decreased glucose level or (2) microorganisms seen on Gram stain or (3) colonization of catheter tip [25]. A large prospective study comparing 146 silvers-impregnated EVD with 188 AI-EVD and 161 unprocessed EVD, found no difference in the incidence of CSF infection [26]. A meta-analysis of one RCT and four cohort studies (two retrospective and two prospective) for a total of 943 patients found that silver-impregnated EVD were associated with a lower risk of infection (RR = 0.60; 95% CI [0.40–0.90]). The authors also reported that there was no difference in mortality whatever the type of catheter used (RR = 1.17; 95% CI [0.76–1.81]) [27]. In a meta-analysis including one RCT and one prospective study, there was no difference in the incidence of VRI between silver-impregnated EVD and unprocessed EVD (OR = 0.33; 95% CI [0.07–1.69]; *p* = 0.18) [28]. Another meta-analysis of six observational studies found no significant benefice of silver EVD on the rate of VRI (OR = 0.71; 95% CI [0.46–1.08]; *p* = 0.11) [29]. Similarly, in a large meta-analysis of 12 studies comparing silver-impregnated and unprocessed CVC: there was no difference in the rate of colonization (OR = 0.907; 95% CI [0.758–1.087]; *p* = 0.290) and in the rate of catheter-related bloodstream infection (CRBSI) (OR = 0.721; 95% CI [0.476–1.094]; *p* = 0.124) [30]. Clinical results of silver compared to unprocessed EVD are resumed in Table 1.

### Adverse Effect

The toxicity of silver ions is a function of their concentration. Silver toxicity seems to be low in the human body. Chronic exposition or ingestion of silver leads to a deposit in tissue and organs which usually are not life-threatening [31]. In the case of EVD, silver is directly delivered into the cerebral parenchyma and ventricles. An animal study showed a cerebral inflammatory response when the brain was exposed to a silver clip [32]. An experimental study assessing the potential toxic effect of ventricular shunt impregnated with silver nanoparticles showed reassuring results. The concentration of silver ions, in a fluid sampled after crossing the catheter, was negligible, thus, limiting the potentiality of adverse effects [23]. Another experimental study on silicone catheters impregnated with silver nanoparticles inserted in mice found that most of the silver remained on the catheter (approximately 16% of the silver was released after ten days). There was no accumulation of silver in the major organs. However, silver accumulated locally in the tissue surrounding the catheter, but its concentration remained far below the toxic level in humans [33]. Clinical results are also reassuring with no reported adverse effect linked to the accumulation of silver ions in organs and tissue [34].

## 5. Antibiotic Impregnated EVD

Two types of AI-EVD are used in clinical practice. Both are made of silicon elastomer. The first one is impregnated with a combination of rifampicin and minocycline (VentriClear^TM^) and the second one is with a combination of rifampicin and clindamycin (Bactiseal^TM^). The last one is the most studied device in the literature. Rifampicin has a broad-spectrum activity against GPC, some GNR, and intracellular bacteria. Its action is mediated through the inhibition of the bacterial DNA-dependent RNA polymerase [35]. It has also been widely used during biomaterial or implant infection because of its activity against Staphylococci-related biofilm. It is usually not advised to use it in monotherapy because of the associated risk with the emergence of resistance [36]. Clindamycin has activity against GPC (*Staphylococci*, *Streptococci*) and most anaerobes. It has no activity against aerobic GNR. Clindamycin inhibits bacterial protein synthesis by reversibly binding the 50S ribosomal subunits [37]. Its activity against biofilm is poor, experimentally [38]. Minocycline is a second-generation tetracycline antibiotic with activity against GPC, GNR (including *Acinetobacter baumanii*), and intracellular bacteria. Minocycline inhibits protein synthesis by binding the 30S ribosomal subunit. Its lipophilic properties allow it to easily cross cellular membranes and the blood-brain barrier [39].

## 6. In Vitro Assessment of Antibiotic Impregnated EVD Efficacy

In 1989, Bayston et al. described a process of impregnation of VS made in silicon with five different antibiotics previously selected because they adequately cross the silicone elastomer, resist the sterilization process, and exhibit optimal activity against GPC [40]. The impregnation process consisted of the immersion of the catheter in a solution of each antibiotic (single or in combination) and chloroform to give a concentration of 0.2% (*w*/*v*). Then, they were challenged three times with a 1 mL solution of *S. epidermidis*, at a concentration of 10^7^ to 10^8^ cfu/mL. The results showed that all of the catheters processed with a single antibiotic were ineffective to prevent bacterial colonization (with the exception of the rifampicin one). Catheters impregnated with rifampicin and clindamycin at a concentration of 0.2% (*w*/*v*) remained uncolonized after the three bacterial challenges [40]. Thus, they were selected for the development of the Bactiseal^TM^ VS and EVD. The Bactiseal^TM^ AI-EVD are impregnated with rifampicin and clindamycin at a concentration of 0.25 to 0.7 mg/g and 0.85 to 1.54 mg/g, respectively [41]. The total dose released by the catheter over time remained below the concentration measured after a single intravenous dose of each antibiotic limiting the risk of side effects [41]. In an in vitro model of antibiotics impregnated VS (AI-VS) was continuously perfused by a solution and challenged with a different strain of *Staphylococci* at a concentration of 10^8^ cfu/mL (the exposition to bacteria range from 5 min to 1 h and was repeated every 14 days), Bayston et al. showed that AI-VS prevent bacterial colonization (assessed by standard culture) by up to 28 days and up to 42 days for *S. epidermidis* and *S. aureus* strains, respectively, while unprocessed catheters were colonized within 48 h. When a conditioning film (mimicking the in vivo protein deposit resulting from blood and CSF flow through the catheter) was applicated to the catheter layer, the effectiveness of AI-VS was similar [42]. They found that the continuous release of antibiotics does not prevent the adherence of bacteria but killed 100% of bacteria attached to the catheter in 50 h [41]. In a recent study, the in vitro activity of AI-EVD exposed to different mediums (air, saline, saline plus protein, and saline plus lipid medium) was tested. Antimicrobial activity drops quickly according to the medium dwelling notably in presence of a saline and lipid solution with no activity after day 28 for methicillin-resistant *Staphylococcus epidermidis* (MRSE), day 30 for MRSA and day 27 for methicillin-susceptible *Staphylococcus aureus* (MSSA) [43]. The decrease in antimicrobial activity could be related to the elution of the antibiotics according to the flow regiment crossing the catheter [41,44]. Indeed, when studying VentriClear^TM^ EVD, Stevens et al. found that a significant amount of antibiotics were eluted in the fluid crossing the catheter [44].

## 7. In Vivo Assessment of Antimicrobial Activity of AI-EVD

### 7.1. Antimicrobial Activity

Mounier et al. studied in vivo durability of the antimicrobial activity of 65 Bactiseal^TM^ AI-EVD [45]. They assessed the inhibition diameter of AI-EVD tips in contact with three different bacteria strains (MRSE, MRSA, and MSSA at a concentration of 10^4^ and 10^5^ cfu/mL) and the concentration of antibiotics remaining on the catheters after their removal. Inhibition diameters significantly decreased according to the duration of EVD placement and the volume of CSF drained (only for MRSE and MRSA). The activity dropped faster for the external side than the internal one, with no inhibition diameter in 23% (19/65) of all the AI-EVD for a median drainage time of 18 [15,16,17,18,19,20,21,22,23,24,25] days and a median volume of CSF drained of 2450 [1594–3246] mL. There was no correlation between clindamycin concentration and both duration of EVD catheterization and the volume of CSF drained, but the concentration decreased by about 80% only after five days of placement. Conversely, the rifampicin concentration dropped quickly according to duration and volume drained. For half of the AI-EVD analyzed for drug quantification, the concentration of rifampicin was less than one percent of the initial concentration [45]. In another in vivo study regarding AI-VS, authors found that the antimicrobial activity decreased by about 50% of the initial concentration within 10 days then it remained stable until approximately three months (97 days) [46].

### 7.2. Prevention of Bacterial Colonization with AI-EVD

When assessed through standard culture, most studies found that AI-EVD significantly decreases the rate of ventriculostomy-related colonization (VRC) [28,47]. However, standard culture has a poor sensibility to diagnosis catheter colonization when compared to other methods such as scanning electron microscopy (SEM) [48,49]. Ramirez et al. studied in vivo formation of biofilm on both unprocessed and Bactiseal^TM^ AI-EVD with SEM. Biofilm was found on 86% of unprocessed EVD and 67% of AI-EVD (*p* = 0.22). After seven days the prevalence of biofilm was 89% and 88% for unprocessed and AI-EVD, respectively [50]. The culture of EVD tip was positive only in 37.5% (12/32). Similarly, observation of intraparenchymal intracranial pressure transducer (ICPT) on SEM showed a prevalence of mature biofilm of 73% while only five percent of the ICPT were positive in standard culture. The short median duration of the ICPT (4 [3,4,5,6,7] days) suggests that the formation of biofilm occurred quickly after insertion [48]. When studying CVC, similar results were found with a rate of biofilm formation of 100% after a median duration of ten days [51].

## 8. Efficacy of AI-EVD in Clinical Practice

The clinical literature shows conflicting results regarding the efficacy of AI-EVD to prevent VRI. Nonetheless, the use of AI-EVD is recommended in the IDSA guidelines [10]. Three RCTs tested the effectiveness of AI-EVD [47,52,53].

The first one compared the incidence of VRI between AI-EVD (impregnated with rifampicin and minocycline) and unprocessed EVD in 288 patients. VRI was defined as a positive CSF culture. All patients underwent prophylactic and then the maintenance of antibiotics during the time of EVD placement. The incidence of VRI was significantly lower in the AI-EVD than in the unprocessed EVD groups (1.3% and 9.4%; *p* = 0.002; respectively). Catheter colonization was significantly lower in the AI-EVD than in the standard EVD groups (17.9% and 36.9%; *p* < 0.0012, respectively). Interestingly all the microorganisms found in the AI-EVD culture were susceptible to minocycline and all the *staphylococcus* strains were susceptible to rifampicin [47].

The second one compared the incidence of VRI between Bactiseal^TM^ AI-EVD and unprocessed EVD in 184 patients. VRI was defined as positive CSF culture associated with a CSF white cell count >10/mm^3^ a CSF protein level > 0.8 g/L and a CSF/serum glucose ratio < 0.4. In the AI-EVD group, the patients received only prophylactic perioperative antibiotics (ampicillin-sulbactam and ceftriaxone) while in the standard group, the antibiotics were maintained during EVD placement. The incidence of nosocomial infection whatever the site concerned was similar between the two groups. The incidence of VRI was similar between AI-EVD and unprocessed EV (1% and 3%; *p* = 0.282, respectively) [52].

The last one compared the incidence of VRI between Bactiseal^TM^ and unprocessed EVD in 357 patients. Proven VRI was defined as a CSF sample demonstrating a positive Gram stain and culture whereas suspected VRI was defined as a CSF sample demonstrating a negative Gram stain but a positive culture or CSF leukocytosis (White blood cell/Red blood cell CSF count > 0.02). The rate of proven VRI was similar in each group (2.3% and 2.8%; *p* = 1.00, respectively). The rate of suspected VRI was also similar in the two groups (17.5% and 20.4%; *p* = 0.504, respectively) [53].

In a large multicenter prospective cohort, analyzing 495 EVD (whose 188 were AI, 161 unprocessed, and 146 silver-impregnated EVD), there was no association between the catheter type and the incidence of VRI. Only the duration of EVD placement (>8 days; OR = 2.54; 95% CI [1.14–5.7]; *p* = 0.02) and regular sampling were significantly associated with the risk of VRI. The median time to infection was 9 [5,6,7,8,9,10,11,12,13,14,15] days [26]. However, a meta-analysis found a reduction of VRI with AI-EVD. In a pooled analysis of one RCT and three prospective observational studies (for a total of 2332 patients), Sonabend et al. found that AI-EVD significantly decreased the rate of VRI (RR = 0.19; 95% CI [0.07–0.52]; *p* = 0.001) [54]. Wang et al. also found that AI-EVD were associated with a significantly decreased risk of CSF infection in their meta-analysis of the three precited RCTs (for a total of 829 patients) (OR = 0.37; 95% CI [0.21–0.64]; *p* = 0.0004) [28]. Clinical results of AI compared to unprocessed EVD are resumed in Table 2.

### Bacterial Selection and Adverse Effects

Currently there is no convincing evidence that AI-EVD or VS increase the risk of antibiotic resistance or bacterial selection [55]. However, in the first RCT, the organisms responsible for VRI isolated in the unprocessed EVD group were predominantly coagulase-negative *Staphylococci*, whereas those isolated in the AI-EVD group were MRSA, *Enterobacter aerogenes*, and *Enterococcus faecalis* [47]. Wong et al. found a similar rate of resistant opportunistic infection in each group [52]. In their meta-analysis, Konstantelias et al. found a higher risk of infection with MRSA and GNR in patients treated with AI-EVD compared to others [27]. However, another study found no difference in the incidence of MRSA with AI-VS compared to unprocessed shunts [56]. There was no reported local or systemic side effect with AI-EVD or VS currently in the literature [47,52,53]. However, no studies were designed to investigate the potential side effects of these antibiotic-impregnated biomaterials. 

## 9. Discussion

EVD impregnated with antimicrobial agents are an attractive concept to prevent VRI. Until their development, prolonged systemic antibiotics were often used during the time of EVD placement raising concerns about the risk of side effects and resistance selection. The IDSA guidelines recommend the use of AI-EVD although clinical studies showed contrasted results [10]. Interestingly, a large national cohort in the United Kingdom and Ireland (21 neurosurgical units for a total of 495 EVD) found that despite this recommendation only a third of the EVD implanted were AI-EVD [26]. Two of three RCTs found negative results regarding the efficacy of AI-EVD to prevent VRI [52,53]. In all RCT, the median time to infection was similar between each group and around day ten [47,52,53]. In the largest prospective study comparing AI-EVD with silver-impregnated and unprocessed EVD, there were no differences in the rate of VRI. The median time to infection was eleven, eight, and seven days for AI, unprocessed, and silver-impregnated EVD, respectively [26]. Results of the meta-analysis called for the use of AI-EVD despite an important heterogeneity in studies [28,54]. Their results should be interpreted with caution for the following reasons: First, the definition of VRI differs from one study to another and numerous cases of colonization could have been considered as infection. Second, the largest non-randomized study included in the meta-analysis which is in favor of AI-EVD is a before-after study comparing four different periods with the use of standardized protocol and different types of AI-EVD during each period [57]. Third, the largest prospective study showing negative results was not included (because its publication occurred later) [26].

In comparison, clinical studies on the effectiveness of antibiotics impregnated (AI)-CVC showed important discrepancies. A meta-analysis of 11 RCTs comparing AI versus unprocessed CVC showed no difference in the rate of CBSRI [58]. Conversely, a recent Cochrane Review including 60 studies showed a significantly lower rate of CBSRI when AI-CVC was used. However, there was no clinical benefice on mortality and clinical sepsis [59]. It is important to note that most of the studies comparing AI versus unprocessed devices used as primary endpoint a microbiological definition of infection without assessing the clinical status of the patients.

Although in vitro studies reported a prolonged activity of AI-EVD, in vivo results showed a quick drop in the antimicrobial activity related to the CSF flow and medium in which the catheter was inserted [45]. In some cases, there was no activity as soon as day ten, which corresponds to the pic of the incidence of VRI [11,12,45]. It could be linked to a continuous elution of the antibiotic through the flow crossing the catheter [44]. In addition, EVD are continuously challenged by a high density of bacteria composing the head skin microbiota despite optimal disinfection and dressing which only decrease bacterial density for a few hours [60]. This is a major difference with VS which are surgically internalized and thus, exposed to bacteria only during the time of surgery. The in vitro studies were primarily designed to assess the efficacy of AI-VS, but EVD are used in different clinical conditions (acute brain injury, acute local and systemic inflammation, other organs failure, prolonged stay in ICU, and use of systemic antibiotic among other) and the pathways of colonization and infection could be different. All these in vivo variables could explain the difference found in the durability of antimicrobial therapy and in the contrasted clinical results.

The primary goal of AI-EVD is to avoid bacterial adhesion and biofilm formation through a continuous release of antibiotics during the time of EVD placement. Colonization of the catheter is the preclude to infection. The standard diagnosis of VRC is based on the positive culture of EVD tip. Some clinical studies report a lower rate of VRC with AI compared to unprocessed EVD [28,47]. However, most of the bacteria involved in AI-EVD colonization were GPC suggesting a lack of efficacy of the impregnated antibiotics [12,27]. After the first phase of adhesion on biomaterial, bacteria rapidly organized themselves as a biofilm which are more difficult to detect by standard culture [61]. When observed through SEM, the rate of biofilm was almost constant and similar in both types of EVD only a few days after insertion [50]. Incidentally, Bayston et al. showed that AI-EVD cannot prevent bacteria adhesion on biomaterial and that the antimicrobial activity is exerted by killing the bacteria attached. In their protocol, the AI-EVD were challenged by bacterial strain intermittently (*Staphylococcus epidermidis* strain was added to the perfusate at day one, four, 10, and 21) [41]. Only a few minutes could be necessary to lead to an irreversible adhesion of the bacteria to the surface leading to the phenotypic and genotypic changes initiating biofilm formation, and as explicated above, EVD are constantly challenged by a high density of bacteria increasing the risk of adhesion and biofilm formation [62]. Observation of other transdermal devices (such as CVC impregnated or not and ICPT) found similar results [48,51]. It suggests that the natural history of such devices is to be colonized by the bacteria belonging to the skin microbiota and that other factors (not fully elucidated yet) triggered the transition to the infection. Regarding silver-impregnated EVD, the lack of effectiveness could be linked to the silver nanoparticles synthesis and impregnation process. As demonstrated in an experimental study the size of silver nanoparticles was too large (500 nm) and not uniformly distributed along the catheter limiting its biocidal activity [22]. Moreover, the inhibition of bacterial growth and biofilm formation is concentration dependent. When used in vivo numerous factors could influence the concentration of silver nanoparticles released over time, such as the deposit of proteins along or the flow regiment crossing the catheter.

The effect of impregnated EVD on bacterial selection and resistance remained unclear. Some studies suggested that silver and AI-EVD could be associated with a higher rate of VRI caused by GNR or MRSA [27,29,50]. However, a large prospective study found no difference in the rate of infection with GNR between the different types of EVD [26]. In the same way, a recent study found no evidence that impregnated EVD increases the risk of bacterial resistance [55].

## 10. Perspectives

The natural history of transdermal devices seems to be colonized by bacteria belonging to the skin microbiota. Factors triggering the transition from colonization to infection remained unclear. Some authors have raised an interesting hypothesis: Healthy biofilm as found on healthy skin, for example, could be protective whereas, an altered biofilm with a decreased bacterial diversity could lead to infection [63,64]. These new paradigms lead to the development of a new therapeutic approach consisting of replacing pathogenic biofilm with new healthy biofilm (i.e., fecal transplant) [63]. In the future, the identification of bacterial species composing biofilm implicated in VRI and its comparison to “healthy” biofilm will improve our understanding of infection pathways.

## 11. Conclusions

In vivo studies showed that colonization of EVD by bacterial biofilm is almost constant. Clinical studies failed to show a superiority of silver or AI-EVD versus unprocessed EVD to prevent VRI. A better understanding of biofilm dynamics on biomaterial will help us to improve our ability to develop new innovating ways to prevent VRI.

## Figures and Tables

**Table 1:** Effectiveness of silver impregnated compared to unprocessed EVD in clinical practice.

Comparison between Silver Impregnated and Unprocessed EVD				
Years of Publication/Authors	Type of Study	Catheter	Number of Patients	Incidence of VRI/Infection Rate According to the Type of EVD
Keong et al., 2012 [24]	Randomized controlled trial	Silver-impregnated EVD (Silverline^TM^) vs. unprocessed EVD	278 patients (138 vs. 140)	12.3% vs. 21.4% *p* = 0.043
Jakobs et al., 2018 [25]	Randomized controlled trial	Silver-impregnated ELD (Silverline^TM^) vs. unprocessed ELD	48 patients (24 in each group)	4.2% vs. 16.7% *p* = 0.16
Jamjoom et al., 2018 [26]	Prospective cohort	Silver-impregnated EVD (Silverline^TM^) vs. unprocessed EVD	307 patients (146 vs. 161)	13.7% vs. 7.5% *p* = 0.09
Wang et al., 2013 [28]	Meta-analysis (1 RCT, 1 prospective study)	Silver-impregnated EVD (Silverline^TM^) vs. unprocessed EVD	317 patients (157 vs. 160)	OR = 0.33; 95% CI [0.07–1.69]; *p* = 0.18
Konstantelias et al., 2015 [27]	Meta-analysis (1 RCT, 2 prospective, and 2 retrospective studies)	Silver-impregnated EVD (Silverline^TM^) vs. unprocessed EVD	943 patients (491 vs. 452)	RR = 0.60; 95% CI [0.40–0.90]
Atkinson et al., 2016 [29]	Meta-analysis (2 prospective and 4 retrospective studies)	Silver-impregnated EVD (Silverline^TM^) vs. unprocessed EVD	1057 patients (504 vs. 553)	OR = 0.71; 95% CI [0.46–1.08]; *p* = 0.11

ELD: External Lumbar Drain. EVD: External Ventricular Drain. OR: Odds Ratio. RCT: Randomized Controlled Trial. RR: Relative Risk.

**Table 2:** Effectiveness of antibiotic-impregnated compared to unprocessed EVD in clinical practice.

Comparison between Antibiotic Impregnated and Unprocessed EVD				
Years of Publication/Authors	Type of Study	Catheter	Number of Patients	Incidence of VRI/Infection Rate According to the Type of EVD
Zabramski et al., 2003 [47]	Randomized controlled trial	Minocycline/Rifampicin EVD vs. unprocessed EVD	288 patients (149 vs. 139)	1.3% vs. 9.4% *p* = 0.0002
Wong et al., 2010 [52]	Randomized controlled trial	Clindamycin/Rifampicin EVD vs. unprocessed EVD	184 patients (90 vs. 94)	1% vs. 3% *p* = 0.282
Pople et al., 2012 [53]	Randomized controlled trial	Clindamycin/Rifampicin EVD vs. unprocessed EVD	357 patients (176 vs. 181)	2.3% vs. 2.8% *p* = 1.00
Jamjoom et al., 2018 [26]	Prospective cohort	Clindamycin/Rifampicin EVD vs. unprocessed EVD	349 patients (188 vs. 161)	7.4% vs. 7.5% *p* = 1.00
Wang et al., 2013 [28]	Meta-analysis (3 RCT)	Clindamycin/Rifampicin Minocycline/Rifampicin EVD vs. unprocessed EVD	829 patients (415 vs. 414)	OR = 0.37; 95% CI [0.21–0.64]; *p* = 0.0004
Sonabend et al., 2015 [54]	Meta-analysis (1 RCT, 3 prospective studies)	Clindamycin/Rifampicin EVD vs. unprocessed EVD	2332 patients	RR = 0.19; 95% CI [0.07–0.52]; *p* = 0.001

EVD: External Ventricular Drain. OR: Odds Ratio. RCT: Randomized Controlled Trial. RR: Relative Risk.

## Data Availability

Not applicable.
